# Aspirin Derivative 5-(Bis(3-methylbut-2-enyl)amino)-2-hydroxybenzoic Acid Improves Thermotolerance via Stress Response Proteins in *Caenorhabditis elegans*

**DOI:** 10.3390/molecules23061359

**Published:** 2018-06-05

**Authors:** Xiao-Bing Huang, Gui-Sheng Wu, Lei-Yu Ke, Xiao-Gang Zhou, Yue-Hu Wang, Huai-Rong Luo

**Affiliations:** 1 State Key Laboratory of Phytochemistry and Plant Resources in West China, Kunming Institute of Botany, Chinese Academy of Sciences, Kunming 650201, China; hxb@swmu.edu.cn; 2 Key Laboratory for Aging and Regenerative Medicine, Department of Pharmacology, School of Pharmacy, Southwest Medical University, Luzhou 646000, China; wgs@swmu.edu.cn (G.-S.W.); zxg@swmu.edu.cn (X.-G.Z.); 3 Key Laboratory of Medical Electrophysiology of Ministry of Education, Collaborative Innovation Center for Prevention and Treatment of Cardiovascular Disease, Southwest Medical University, Luzhou 646000, China; 4 Key Laboratory of Economic Plants and Biotechnology, and Yunnan Key Laboratory for Wild Plant Resources, Kunming Institute of Botany, Chinese Academy of Sciences, Kunming 650201, China; keleiyu@mail.kib.ac.cn; 5 Southeast Asia Biodiversity Research Institute, Chinese Academy of Sciences, Yezin, Nay Pyi Taw 05282, Myanmar

**Keywords:** aging, aspirin, thermotolerance, lifespan, *Caenorhabditis elegans*

## Abstract

Aging is a major risk factor for many prevalent diseases. Pharmacological intervention to improve the health span and extend the lifespan could be a preventive elixir for aging and age-related diseases. The non-steroid anti-inflammation medicine aspirin was reported to delay aging in *Caenorhabditis elegans* (*C. elegans*) and mice. We are wondering if the analogues of aspirin could also present antiaging activity. Here, we synthesized several aspirin derivatives and investigated their thermotolerance and antiaging effect in *C. elegans*. One of the compounds, 5-(bis(3-methylbut-2-en-1-yl)amino)-2-hydroxybenzoic acid, moderately increased the survival of *C. elegans* under heat stress, but could not extend the lifespan under optimum conditions. This compound could increase the mRNA level of stress response gene *gst-4*, and the mRNA and protein expression level of heat shock protein *hsp-16.2* under heat stress. The failure of activating the transcription factor DAF-16 might explain why this compound could not act as aspirin to extend the lifespan of *C. elegans*. Our results would help further the investigation of the pharmacological activity of aspirin analogues and the relationship between structures and activity.

## 1. Induction

Aging is a deteriorating process from fitness and may eventually lead to many prevalent diseases, such as neurodegenerative disease, cardiovascular disease, cancer, arthritis, and diabetes. Healthcare expenses become a huge burden for both society and aging populations [1]. Therefore, finding out effective strategies to improve the health span would be an important goal in aging research. Pharmacological intervention might be an effective way to improve health span, together with delaying the onset of aging related diseases. Until now, many compounds with antiaging activities in yeast, worms, drosophila, and mice were discovered, such as resveratrol [2,3]; chlorogenic acid [4]; quercetin [5]; the extracts of green and black tea [6], blueberry polyphenols [7], ginkgo biloba [8]; and clinical medicine including metformin [9,10], aspirin [11,12], rapamycin [13], and caffeine [14]. A detailed information of compounds with antiaging activity were reviewed elsewhere [15,16,17]. The possible clinical benefits of these antiaging chemicals for humans are under vigorous research [18].

Aspirin is a small molecule compound with applications for the clinical treatment of pain, fever, and inflammation. Long-term use of aspirin ameliorates the onset of heart attack [19], ischemic stroke [20], colorectal cancer [21], and neurodegenerative diseases [12]. In addition, Aspirin could increase maximum and mean lifespan in yeast, worms, mice, and humans [11,21,22,23,24,25]. Aspirin extends the lifespan and improves thermotolerance through the dietary restriction-like mechanism in *C. elegans* [21,24]. We are wondering if the aspirin analogues could also present antiaging activity.

Studies have demonstrated that the antiaging effect might cause remarkable heat stress improvement [22,26]. The heat stress resistance was reported to present high correlation to longevity [27]. The enhanced expression of heat shock protein HSP-16.2 was well predictable of lifespan extension [28]. Therefore, we synthesized several aspirin analogues and assayed their activity for the enhancement of HSP-16.2 expression. We found one of the compounds, 5-(bis(3-methylbut-2-en-1-yl)amino)-2-hydroxybenzoic acid (**3**), prolonged the survival under thermal stress, while it did not extend the lifespan in permissive conditions.

## 2. Results and Discussion

### 2.1. The Synthesis of Compounds

**Figure 1:**
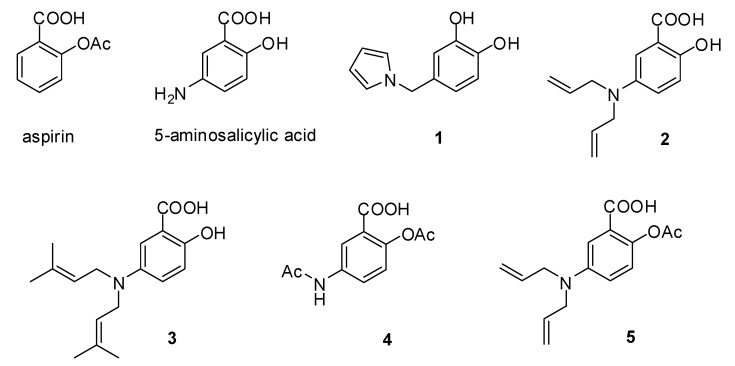
Structures of aspirin, 5-aminosalicylic acid, and synthetic analogues (**1**–**5**).

Five analogues (**1**–**5**, Figure 1) of aspirin were synthesized. A condensation of 3,4-dimethoxybenzaldehyde and *trans*-4-hydroxy-l-proline catalyzed by AcOH yielded a pyrrole derivative [29]. The pyrrole derivative was demethylated using BBr_3_ to give 4-((1*H*-pyrrol-1-yl)methyl)benzene-1,2-diol (**1**) [30]. 5-Aminosalicylic acid (5-ASA) was alkylated using allyl bromide and 3,3-dimethylallyl bromide to afford 5-(diallylamino)-2-hydroxybenzoic acid (**2**) and 5-(bis(3-methylbut-2-enyl)amino)-2-hydroxybenzoic acid (**3**), respectively. 5-ASA and **2** were acetylated to yield 5-acetamido-2-acetoxybenzoic acid (**4**) and 2-acetoxy-5-(diallylamino)benzoic acid (**5**), respectively. Materials and methods showed more details.

### 2.2. Thermotolerance Enhancement Activity of Aspirin Analogues Were Assayed in C. elegans

**Figure 2:**
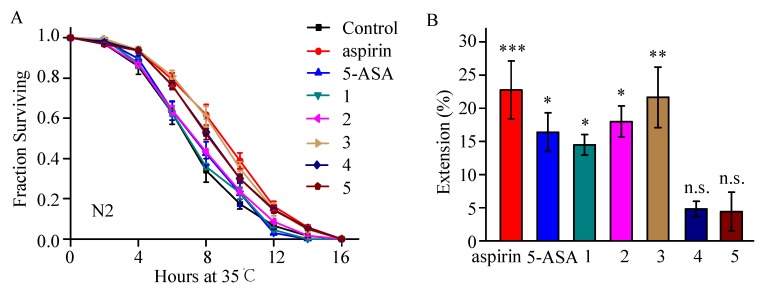
The thermotolerance activity of the analogues of aspirin. (**A**) Figures showed the survival rate of control and worms treated with different compounds in thermotolerance in 35 °C. Error bars represent mean ± SEM; (**B**) Extension was shown in mean value of three independent experiments, and error bars represent SEM. * *p* < 0.05, ** *p* < 0.01, and *** *p* < 0.001; n.s. is not significant. At least 40 worms were used in each group, and three biological repetitions were performed in every assay.

The physiological performances often decrease in nematode accompanying aging, including body movement, pharyngeal pumping, egg-laying, and stress resistance [31]. To investigate whether the purchased aspirin analogue 5-ASA and the synthetic compounds (**1**–**5**) could delay age-related decline of phenotypes, we measured the heat stress resistance of worms in 35 °C after being treated with five days of each compounds (aspirin, 5-ASA, **1**–**5**), and aspirin was used as a positive control. Among these compounds, 5-ASA is a component in the treatment of ulcerative colitis and inflammatory bowel diseases [32]. Our results showed that 5-ASA slightly increased the heat stress resistance in worms (*p* < 0.05, Figure 2A,B). Compounds **1** and **2** also faintly increased the heat stress resistance in worms (*p* < 0.05, Figure 2A,B), while compounds **4** and **5** did not (*p* > 0.05, Figure 2A,B), suggesting that acetyl might not be necessary for the stress resistance enhancement. Among these synthetic compounds, we found compound **3** could increase survival in heat stress by up to 22% (*p* < 0.01, Figure 2A,B), similar to the effect of aspirin (*p* < 0.001, Figure 2A,B), indicating that compound **3** might have an antiaging effect, as with aspirin.

### 2.3. Compound ***3*** Barely Extends Lifespan under Normal Culture Condition

**Figure 3:**
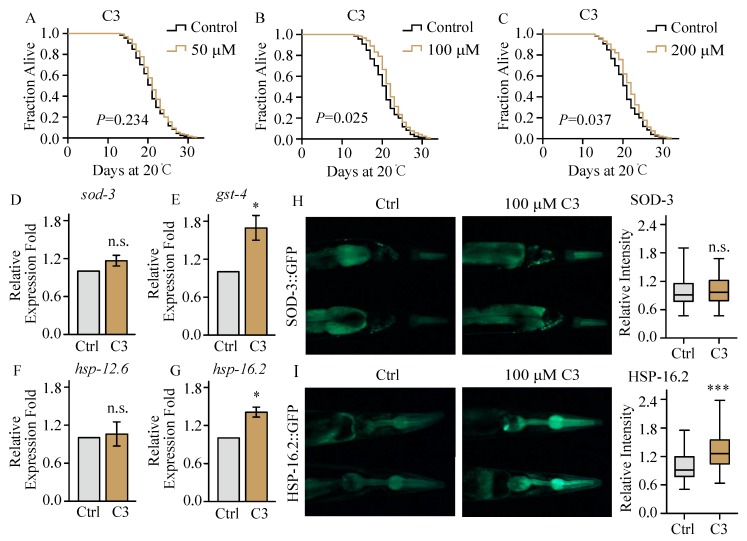
Compound 3 upregulated the expression level of stress response proteins GST-4 and HSP-16.2. (**A**–**C**) The survival curves of wild type worms rose at 20 °C on NGM plates containing various concentrations of compound **3**. Lifespan was analyzed by Kaplan-Meier, and *p* values were calculated by log-rank test. For lifespan assay, at least 80 worms were used in each group, and three biological repetitions were performed; (**D**–**G**) The mRNA expressions level of *sod-3*, *gst-4*, *hsp-12.6,* and *hsp-16.2* under the treatment of compound **3**. Figures show the mean value of three independent experiments, and error bars represent SEM. P values were calculated by two-tailed *t*-test, * *p* < 0.05; n.s. is not significant; (**H**–**I**) SOD-3::GFP and HSP-16.2::GFP were induced by compound **3** and observed under fluorescent microscopy. For HSP-16.2::GFP expression assay, age synchronized young adults with HSP-16.2::GFP were transferred to compound **3** treatment for 48 h, then shifted to 35 °C for 2 h, and followed by recovery for 24 h. At least 40 worms were used in each group, and three biological repetitions were used in assays. Relative GFP fluorescence intensity was calculated by Image J. For box-and-whisker plots; whiskers show minima and maxima within 1.5 interquartile ranges (IQR). P values were calculated by two-tailed *t*-test, *** *p* < 0.001.

With prolonged survival in thermal stress, we determined the lifespan of worms treated with compound **3** in 20 °C. Worms were treated with compound **3** in L4 larvae or young adults and transferred to fresh plates with or without compounds every other day. Worms were observed every day and scored as dead when they did not respond to a mechanical stimulus. Our results showed that 50 μM of compound **3** could not extend the lifespan of worms (*p* > 0.05, Figure 3A). When worms were treated with 100 μM and 200 μM of compound **3**, the mean lifespan could be slightly extended by 4.0% (*p* < 0.05, Figure 3B) and 4.2% (*p* < 0.05, Figure 3C), respectively. These findings indicated that compound **3** could barely extend the lifespan of *C. elegans*, nor in a dose-dependent manner.

### 2.4. Compound ***3*** Increases the Expression of Stress Response Proteins in C. elegans under Heat Stress

Many transcription factors that modulate stress response and aging have been identified in the nematode, like DAF-16, HSF-1, and SKN-1 [33]. Worms under adverse conditions, such as food depletion, overcrowding, high temperature, or oxidative stress, DAF-16 would be translocated to the nucleus and activate the expression of its targeted genes [34,35,36]. Therefore, we measured whether compound **3** could activate the nuclear localization of DAF-16. Worms of TJ356 (DAF-16::GFP) treated with compound **3** or mock were transferred to short thermal stress at 37 °C for 20 min. We found that treatment of compound **3** did not exhibit accumulation of DAF-16 in the nucleus either at 12 h or 24 h after thermal stress (data not shown). Enhancing the activity of the FOXO transcription factor DAF-16 together with increased expression of its targeted gene encoding mitochondrial superoxide dismutase (*sod-3*) could significantly increase lifespan and stress tolerance [37]. To further investigate if compound **3** could regulate the activity of DAF-16, we detected the expression of *sod-3* in mRNA and protein level. Results showed that compound **3** did not increase the *sod-3* expression level of mRNA (*p* > 0.05, Figure 3D), nor the fluorescence intensity of SOD-3::GFP (*p* > 0.05, Figure 3H), suggesting that compound **3** did not regulate the activity of DAF-16 to enhance thermal tolerance.

The heat shock transcription factor HSF-1 and Nrf2-like xenobiotic and oxidative stress response factor SKN-1 regulate aging and stress response in worms [38]. HSF-1 and SKN-1 induce transcriptional and translational response to intense thermal stress [39]. To explore whether compound **3** regulates the activity of HSF-1 or SKN-1, we determined the mRNA level of their targeted stress response related genes under treatment of compound **3**. Our results showed that compound **3** increased the mRNA expression level of SKN-1 targeted gene *gst-4* (*p* < 0.05, Figure 3E) and HSF-1 targeted gene *hsp-16.2* under heat stress (*p* < 0.05, Figure 3G). To further confirm the induction of stress response, proteins were increased after compound **3** treatment *in vivo*, and transgenic worms with HSP-16.2::GFP and GST-4::GFP were used. Age synchronized young adults with HSP-16.2::GFP were treated with compound **3** for 48 h. Then, worms were shifted to 35 °C for 2 h and recovered for 24 h [40]. For quantification of GST-4, age-synchronized young adults of GST-4::GFP of L1 larvae were treated with compound **3** for 72 h [41]. The GFP fluorescence of worms was directly observed by fluorescence microscope. Results showed that compound **3** significantly increased the expression of HSP-16.2::GFP (*p* < 0.001, Figure 3I), while we failed to measure the expression of *gst-4* due to the weak fluorescence intensity of *gst-4p::GFP*.

### 2.5. Discussion

Our results showed that among the six aspirin analogues, compound **3** could modestly increase the survival of *C. elegans* under thermal stress but could not extend the lifespan. Numerous mechanisms could induce cell stress responses, such as decreased insulin signaling [42], reduced germline signaling [43], activated mitochondrial unfolded protein response [44], endoplasmic reticulum stress [45], and upregulated heat stress response [46]. All these signals could activate crucial stress regulating transcription factors, such as FOXO-like DAF-16, Nrf2-like SKN-1, and heat shock transcription factor HSF-1 to induce the expression of stress response and further extend the lifespan [47]. We found that compound **3** could not activate DAF-16, while it could increase the mRNA expression level of SKN-1targeted gene *gst-4*, and it could also increase the mRNA level of HSF-1 targeted gene *hsp-16.2* and its encoding protein level under heat stress.

Transient heat shock could activate the *hsp-16.2* expression, and the increased expression of *hsp-16.2* is highly correlated with subsequent longevity [28,48]. Previous study showed that there was no detectable HSP16 in young wild type worms and *age-1(hx546)* worms at 20 °C, while after heat shock, over accumulation of HSP16 in *age-1(hx546)* strain was observed throughout the lifespan of adult worms and conferred to longevity extension [49]. Our results showed that compound **3** increased the expression of *hsp-16.2* after heat shock without lifespan extension. It might be suggested that compound **3** could not activate heat shock factors without pretreatment (transient heat shock). Studies showed that high-dose of aspirin increased the expression of heat shock protein *in vivo* even without heat treatment [50]. In our lifespan assays, we transferred late L4 larvae or young adults to NGM plates containing different concentrations of compound **3** at 20 °C for the whole life of worms. Therefore, we speculated compound **3** could not activate *hsp-16.2* at 20 °C during lifespan assays, while activated *hsp-16.2* after heat shock to protect the damage of worms from heat stress. In addition, aspirin could activate the crucial lifespan regulator DAF-16, but not compound **3**, suggesting the underlying mechanism that compound **3** could not extend the lifespan of *C. elegans*.

## 3. Materials and Methods

### 3.1. Chemistry and Synthesis

All the reagents were used without further purification unless otherwise specified. Solvents were dried and redistilled prior to use according to the standard method. ^1^H- and ^13^C-NMR spectra were collected on a Bruker AM-400, DRX-500, and Avance III-600 spectrometers (Bruker Bio-Spin GmbH, Rheinstetten, Germany) with TMS as an internal standard. ESI-MS and HR-ESI-MS analyses were performed on an API QSTAR Pulsar 1 spectrometer (Applied Biosystems/MDS Sciex, Foster City, CA, USA). HR-EI-MS were performed on a Waters AutoSpec Premier p776 spectrometer (Waters, Millford, MA, USA). Silica gel G (80–100 and 300–400 mesh, Qingdao Meigao Chemical Co., Ltd., Qingdao, China) and C_18_ silica gel (40–75 μm, Fuji Silysia Chemical Ltd., Aichi, Japan) were used for column chromatography, and silica gel GF_254_ (Qingdao Meigao Chemical Co., Ltd., Qingdao, China) was used for preparative TLC as precoated plates. TLC spots were visualized under UV light at 254 nm and by dipping into 5% H_2_SO_4_ in alcohol followed by heating. Semipreparative HPLC was performed on an Agilent 1200 series pump (Agilent Technologies, Santa Clara, CA, USA) equipped with a diode array detector and an Agilent Zorbax SB-C_18_ column (5.0 μm, *φ* 9.4 × 250 mm) or a Welch Ultimate AQ-C_18_, column (5 μm, *φ* 4.6 × 300 mm, Welch Materials Inc., Shanghai, China).

#### 3.1.1. 4-((1*H*-Pyrrol-1-yl)methyl)benzene-1,2-diol (**1**)

A solution of *trans*-4-hydroxy-l-proline (1.18 g, 9.03 mmol) and AcOH (35 µL) in DMF (18 mL) was stirred at 160 °C. 3,4-Dimethoxybenzaldehyde (1 g, 6.02 mmol) in 6 mL of DMF was added dropwise during 30 min. The reaction mixture was stirred for additional 10 min. It was purified by flash column chromatography on silica gel (EtOAc-hexanes, 0% to 5%, 60 min) to give 1-(3,4-dimethoxybenzyl)-1*H*-pyrrole (0.87 g, 4.00 mmol, 66%) as a colorless oil; ^1^H-NMR (CDCl_3_, 400 MHz) *δ* 6.82 (1H, d, *J* = 8.1 Hz), 6.71 (1H, dd, *J* = 8.1, 2.0 Hz), 6.68 (2H, t, *J* = 2.1 Hz), 6.65 (1H, d, *J* = 2.0 Hz), 6.18 (2H, t, *J* = 2.1 Hz), 5.00 (2H, s), 3.86 (3H, s), 3.82 (3H, s).

To a stirred solution of 1-(3,4-dimethoxybenzyl)-1*H*-pyrrole (0.87 g, 4.00 mmol) in CH_2_Cl_2_ (40 mL) at −78 °C was added a solution of BBr_3_ (8 mL, 8.00 mmol) in CH_2_Cl_2_ (1 M) dropwise. The mixture was warmed to room temperature, stirred for 2 h, and poured into ice-water (100 mL). The organic phase was separated and the solvent was removed. The crude product was purified by flash silica gel chromatography with MeOH/CH_2_Cl_2_ (0% to 5%, 60 min) as eluent to afford **1** (0.41 g, 2.17 mmol, 54%) as a white solid. ^1^H-NMR (CDCl_3_, 400 MHz) *δ* 6.80 (1H, d, *J* = 8.0 Hz), 6.67 (2H, t, *J* = 2.1 Hz), 6.62 (1H, dd, *J* = 8.0, 2.0 Hz), 6.60 (1H, d, *J* = 2.0 Hz), 6.18 (2H, t, *J* = 2.1 Hz), 5.10 (2H, s); HR-EI-MS *m*/*z* 189.0790 [M]^+^ (calcd. for C_11_H_11_NO_2_, 189.0790).

#### 3.1.2. 5-(Diallylamino)-2-hydroxybenzoic acid (**2**)

To a solution of 5-aminosalicylic acid (0.5 g, 3.27 mmol) and triethylamine (0.91 mL, 6.53 mmol) in dried DMF (10 mL) was added allyl bromide (0.56 mL, 6.53 mmol) at room temperature under a nitrogen atmosphere. The reaction mixture was stirred for 4 h at room temperature. Ice chips were added to the reaction mixture, and then solvent was removed in vacuo. The residue was purified by column chromatography (CHCl_3_/MeOH, 3:1) and recrystallized from MeOH to give **2** (0.17 g, 0.73 mmol, 22%) as a white solid; ^1^H-NMR (DMSO-*d*_6_, 400 MHz) *δ* 7.04 (1H, d, *J* = 2.0 Hz), 6.95 (1H, br d, *J* = 8.8 Hz), 6.78 (1H, d, *J* = 8.8 Hz), 5.81 (2H, m), 5.12 (4H, m), 3.83 (4H, d, *J* = 3.5 Hz); ^13^C-NMR (DMSO-*d*_6_, 100 MHz) *δ* 172.1 (C), 152.8 (C), 141.2 (C), 134.6 (CH × 2), 122.0 (CH), 117.4 (CH), 116.2 (CH_2_ × 2), 112.8 (CH), 112.6 (C), 53.1 (CH_2_ × 2); ESI-MS *m*/*z* 234 [M + H]^+^; HR-ESI-MS *m*/*z* 234.1126 [M + H]^+^ (calcd. for C_13_H_16_NO_3_, 234.1130).

#### 3.1.3. 5-(Bis(3-methylbut-2-enyl)amino)-2-hydroxybenzoic acid (**3**)

To a solution of 5-aminosalicylic acid (1.5 g, 9.79 mmol) and triethylamine (2.73 mL, 19.58 mmol) in dried DMF (30 mL) was added 3,3-dimethylally bromide (2.55 mL, 19.58 mmol) at room temperature under a nitrogen atmosphere. The reaction mixture was stirred for 4 h at room temperature. Ice chips were added to the reaction mixture, and then solvent was removed in vacuo. The residue was purified by column chromatography (CHCl_3_/MeOH, 3:1) and recrystallized from MeOH to give **3** (0.95 g, 3.28 mmol, 34%) as a white solid; ^1^H-NMR (CD_3_OD, 500 MHz) *δ* 7.93 (1H, br s), 7.57 (1H, br d, *J* = 8.5 Hz), 7.05 (1H, d, *J* = 8.5 Hz), 5.16 (2H, br s), 4.19 (4H, d, *J* = 6.0 Hz), 1.71 (6H, s), 1.59 (6H, s); ^13^C-NMR (DMSO-*d*_6_, 100 MHz) *δ* 172.6 (C), 163.7 (C), 146.0 (C), 130.4 (C), 129.3 (CH), 126.0 (CH), 119.9 (CH), 114.1 (CH × 2), 56.7 (CH_2_ × 2), 26.0 (CH_3_ × 2), 18.3 (CH_3_ × 2); ESI-MS *m*/*z* 290 [M + H]^+^, 312 [M + Na]^+^; HR-ESI-MS *m*/*z* 290.1753 [M + H]^+^ (calcd. for C_17_H_24_NO_3_, 290.1756).

#### 3.1.4. 5-Acetamido-2-acetoxybenzoic acid (**4**)

To a solution of 5-aminosalicylic acid (1 g, 6.53 mmol) in Ac_2_O (4.8 mL) was added K_2_CO_3_ (2.7 g). The reaction mixture was stirred for 30 min at 60 °C. Then, 40 mL ice water with pH 3–4 was added. The mixture was filtrated and washed with ice water. The residue was dried to yield **4** as a white powder (522 mg, 2.20 mmol, 34%); ^1^H-NMR (CD_3_OD, 400 MHz) *δ* 8.14 (1H, d, *J* = 2.7 Hz), 7.79 (1H, dd, *J* = 8.7, 2.7 Hz), 7.03 (1H, d, *J* = 8.7 Hz), 2.22 (3H, s), 2.09 (3H, s); ESI-MS *m*/*z* 260 [M + Na]^+^; HR-ESI-MS *m*/*z* 260.0532 [M + Na]^+^ (calcd. for C_11_H_11_NNaO_5_, 260.0535).

#### 3.1.5. 2-Acetoxy-5-(diallylamino)benzoic acid (**5**)

To a solution of **2** (0.15 g, 0.65 mmol) in Ac_2_O (1 mL) was added K_2_CO_3_ (0.14 g). The reaction mixture was stirred for 30 min at 60 °C. Then, 10 mL ice water with pH 3–4 was added. The mixture was filtrated and washed with ice water. The residue was dried to yield **5** as a white solid (8 mg, 0.029 mmol, 4%); ^1^H-NMR (CD_3_OD, 400 MHz) *δ* 7.32 (1H, s), 6.92 (2H, overlapped), 5.89 (2H, m), 5.20 (4H, m), 3.99 (4H, m), 2.25 (3H, s); ESI-MS *m*/*z* 298 [M + Na]^+^; HR-ESI-MS *m*/*z* 298.1059 [M + Na]^+^ (calcd. for C_15_H_17_NNaO_4_, 298.1055).

### 3.2. Culture Conditions and Worm Strains

Strains were obtained from Caenorhabditis Genetics Center (CGC) and maintained at appropriate temperature. Strains used in this study were N2 (Bristol, wild type), CF1553 *(muIs84 [Psod-3:GFP, rol-6])*, CL2070 *(dvIs70 [Phsp-16.2::GFP, rol-6])*, CL2166 *(dvIs19 [Pgst-4p::GFP])*, and TJ356 *daf-16(zls356)IV*. All strains were maintained and grown on NGM plates seeded with *Escherichia coli* OP50. Compounds were dissolved in DMSO for storage and resolved in PBS to a serial of concentration before use. After adding the compound to the NGM plates, the final DMSO concentration was 0.1%. The negative control group had the same concentration of dissolvent with the treatment group. NGM plates containing compounds were equilibrated overnight before use.

### 3.3. Thermotolerance Assay

For thermotolerance assay, L4 larvae or young adults were transferred to NGM plates with or without compounds; worms were transferred to fresh plates every day. At the day five, worms were incubated at 35 °C and monitored for their survival [7,27]. Animals were scored as dead when they did not respond to the gentle touch with platinum wire pick every two hours. At least 40 worms were used for each experiment.

### 3.4. Lifespan Assay

Strains were cultured on fresh NGM plates for 2–3 generations without starvation, and lifespan analysis was conducted at 20 °C, unless otherwise stated. Late L4 larvae or young adults were transferred to NGM plates containing inactivated OP50 (65 °C for 30 min) and 20 μM of FUdR to inhibit the growth of progeny [26]. The day of L4 larvae or young adults transferred to a NGM plate was defined as the test day 0. Then, worms were transferred to fresh plates every other day. Worms were censored if they crawled off the plate, displayed extruded internal organs, or died because of hatching progeny inside the uterus. Worms that did not respond to a mechanical stimulus were scored as dead.

### 3.5. Green Fluorescent Protein Quantification and Visualization

For the quantification of HSP-16.2 and SOD-3, age synchronized CL2070 *(dvIs70 [Phsp-16.2::GFP, rol-6])* and CF1553 *(muIs84 [Psod-3::GFP, rol-6])* of adult day one worms were transferred to compounds treated and untreated plates for 48 h. Worms were shifted to 35 °C for 2 h and then allowed to recover for 24 h [40]. After recovery, worms were imaged and assessed under fluorescence microscope (Olympus, IX51). For the quantification of GST-4, age synchronized CL2166 *(dvIs19 [Pgst-4p::GFP])* of L1 larvae were transferred to compounds treated and untreated plates for 72 h [41]. The GFP fluorescence of worms was directly observed by fluorescence microscope (Olympus, IX51). For GFP quantification, images were analyzed by Image J.

### 3.6. DAF-16::GFP Localization Assay

For each experiment, at least 30 worms of transgenic strain TJ356 *daf-16(zls356)IV.* were used to analyze the localization of DAF-16::GFP in the same condition. L4 larvae were transferred to the plates with or without compounds. Worms were cultured for 12 h and 24 h at 20 °C [4]. Before observation, the worms for positive control were transferred to short thermal stress at 37 °C for 20 min. The location of DAF-16::GFP signal was monitored using a fluorescent microscope system (Olympus, IX51). The accumulation of fluorescent signal in nuclei was scored as described preciously [51].

### 3.7. Gene Expression Assay

About 2000 synchronized young adult worms were transferred to NGM plates with or without compounds and culture at 20 °C for 24 h. Pretreated worms were shifted to 35 °C for 2 h and then allowed to recover for 12 h. Total RNA was extracted using RNAiso Plus (Takara) and converted to cDNA using High Capacity cDNA Reverse Transcription Kit (Applied Biosystems). The qRT-PCR reactions were performed using Power SYBR Green PCR Master Mix (Applied Biosystems) and ABI 7500 system [24]. The relative expression level of genes was carried out using 2^−∆∆CT^ method and normalized to the expression of gene *cdc-42*. The following primes were used:

*cdc-42* F 5′-CTGCTGGACAGGAAGATTACG-3′; R 5′-CTCGGACATTCTCGAATGAAG-3′;
*sod-3* F 5′-AGCATCATGCCACCTACGTGA-3′; R 5′-CACCACCATTGAATTTCAGCG-3′;
*gst-4* F 5′-TCCGTCAATTCACTTCTTCCG-3′; R 5′-AAGAAATCATCACGGGCTGG-3′;
*hsp-12.6* F 5′-GTGATGGCTGACGAAGGAAC-3′; R 5′-GGGAGGAAGTTATGGGCTTC-3′;
*hsp-16.2* F 5′-CTGCAGAATCTCTCCATCTGAGTC-3′; R 5′-AGATTCGAAGCAACTGCACC-3′.

### 3.8. Statistical Analyses

All assays were repeated in at least three independent trials. Statistical analyses were carried out using SPSS 19.0 packages. Data were shown in mean ± SEM. Figures were drawn by OriginPro 8. One-way ANOVA followed by Dunnett (equal variances assumed) or Dunnett’s T3 (equal variances not assumed) *post-hoc* test to determine difference between control group and compounds treated groups in thermotolerance assay. Kaplan-Meier lifespan analysis was carried out, and *p* values were calculated using log-rank test in lifespan assay. Two-tailed student’s *t*-test was used to compare two groups in genes expression and fluorescent protein quantification. *p* < 0.05 was considered as significant.

## 4. Conclusions

We synthesized five aspirin analogues. Among them, compound **3** moderately enhanced the thermal stress tolerance. Compound **3** could increase the expression of *hsp-16.2* under heat stress, but could not activate *daf-16*, which might be the underlying mechanism that prevented compound **3** from extending the lifespan of *C. elegans*. Our results would be helpful for further investigating the pharmacological activity of aspirin analogues and the relationship between their structures and biological activities.
